# Substitution of Mannan-Binding Lectin (MBL)-Deficient Serum With Recombinant MBL Results in the Formation of New MBL/MBL-Associated Serine Protease Complexes

**DOI:** 10.3389/fimmu.2018.01406

**Published:** 2018-06-27

**Authors:** Mischa P. Keizer, Angela Kamp, Gerard van Mierlo, Taco W. Kuijpers, Diana Wouters

**Affiliations:** ^1^Department of Immunopathology, Sanquin Blood Supply, Division Research and Landsteiner Laboratory of the Academic Medical Center, University of Amsterdam, Amsterdam, Netherlands; ^2^Department of Pediatric Hematology, Immunology and Infectious Diseases, Emma Children’s Hospital, AMC, University of Amsterdam, Amsterdam, Netherlands; ^3^Department of Blood Cell Research, Sanquin Blood Supply, Division Research and Landsteiner Laboratory of the AMC, University of Amsterdam, Amsterdam, Netherlands

**Keywords:** recombinant MBL, MBL-associated serine protease, redistribution, mannan-binding lectin–MBL-associated serine protease complexes, size-exclusion chromatography

## Abstract

The lectin pathway (LP) of complement activation depends on the activation of the MBL-associated serine proteases (MASPs) circulating in complex with mannan-binding lectin (MBL). MBL deficiency is the most common complement deficiency and has been associated with several pathological conditions. As we had previously shown, plasma-derived MBL (pdMBL) contains pre-activated MASPs that upon *in vivo* pdMBL substitution results in restoration of MBL concentrations but no LP functionality due to immediate inactivation of pdMBL–MASP complexes upon infusion. In this study, we analyzed MBL-sufficient and -deficient serum by size-exclusion chromatography for complexes of LP activation. In both sera, we identified non-bound free forms of MASP-2 and to lesser extent MASP-1/3. After addition of recombinant MBL (rMBL) to MBL-deficient serum, these free MASPs were much less abundantly present, which is highly suggestive for the formation of high-molecular complexes that could still become activated upon subsequent ligand binding as shown by a restoration of C4-deposition of MBL-deficient serum. Ficolin (FCN)-associated MASPs have been described to redistribute to ligand-bound MBL, hereby forming new MBL/MASP complexes. However, reconstitution of MBL-deficient serum with rMBL did not change the relative size of the FCN molecules suggestive for a limited redistribution in fluid phase of already formed complexes. Our findings demonstrate that rMBL can associate with free non-bound MASPs in fluid phase while preserving full restoration of LP functionality. In contrast to pdMBL products containing pre-activated MASPs which become inactivated almost immediately, these current data provide a rationale for substitution studies using rMBL instead.

## Introduction

The complement system is an intricate and subtle cascade comprising more than 50 soluble and cell-bound proteins to defend against a wide range of bacterial and fungal pathogens. The complement system has an array of different functions, which includes opsonization and lysis of pathogens, elimination of immune complexes, and stimulation and chemotaxis of leukocytes (1, 2).

In general, the complement system is divided into three activating pathways, which all converge into the so-called terminal pathway. The antibody-dependent classical pathway (CP) is activated upon the binding of C1q to immune complexes. Upon binding of mannan-binding lectin (MBL) (or other collectins such as CL-L1 or CL-K1), or one of the three ficolins (FCNs), to pathogen-specific carbohydrates, the MBL-associated serine proteases (MASPs) are activated resulting in activation of the lectin pathway (LP). The alternative pathway is spontaneously activated on surfaces that lack complement regulatory proteins and can act as an amplification loop for both the CP and the LP. All three activating pathways converge at the level of C3 and can subsequently activate the terminal pathway to form the membrane attack complex for complement-mediated lysis of target cells.

Several LP pattern-recognition molecules (PRMs) have been identified. Most important are MBL and the so-called FCNs (3). In serum, the basic unit of MBL (a trimer of 32 kDa polypeptide chain) oligomerizes to form higher-order molecules ranging from dimers to hexamers (650 kDa) and higher (4, 5). MBL circulates in complex with the inactive zymogens of MASPs (4). The different higher-order oligomeric forms of MBL may have a different composition of MASPs, possibly combining different MASPs in one complex (6, 7). There are two major MASPs: i.e., MASP-2 and MASP-1. The 74-kDa MASP-2 is auto-activated upon ligand binding of the PRMs, and the activation is greatly increased in the presence of 77-kDa MASP-1 (8, 9). MASP-3 is a splice variant of the same *MASP1/3* gene with catalytic activity and has an important role in the activation of factor D (10). Different MASPs can be present within a single oligomeric MBL–MASP complex (6, 8). The role of MASP-1 in the activation of MASP-2 suggests that a heterocomplex of these MASPs with MBL is required for LP activation (8, 9). Alternatively, smaller MBL–MASP complexes could bind in close vicinity to allow cross-activation of MASP-2 by MASP-1 (6).

Mannan-binding lectin deficiency is a common complement deficiency. Depending on the definition up to 20% of the human population is affected, and MBL deficiency has been associated with several diseases (11–13). MBL deficiency in different vulnerable patient groups, including pediatric oncology patients (12), neonates (14), and patients with cystic fibrosis (15), has shown an increased severity and frequency of infections. Earlier MBL-substitution trials with plasma-derived MBL (pdMBL) in MBL-deficient individuals have shown to be safe, without any major adverse event reported (11). Although MBL levels were restored, LP functionality remained unexpectedly low following *in vivo* MBL substitution (16). This was found to be due to immediate interaction of inhibitors, such as C1-inhibitor (C1-INH), in the recipient blood with the pre-activated MBL–MASP complexes in the pdMBL product (17). Substitution with rMBL would restore the C4-deposition of MBL-deficient serum upon substitution (18), without the rapid inactivation seen in pdMBL. For this reason, there is renewed interest in the use of rMBL.

In this study, we analyzed the composition and size of LP-activating complexes in MBL-sufficient, MBL-deficient, and rMBL-reconstituted deficient serum in detail to determine whether rMBL associates with MASPs to act as effective LP-activating complexes upon MBL substitution.

## Materials and Methods

### Reagents

Mouse monoclonal antibodies (mAbs) against MBL (e.g., αMBL-1; murine IgG1) were generated at our department, and the properties have been described before (19–21). mAbs against MASP-1/3 (4H2A9; murine IgG1), MASP-2 (8B5; murine IgG1), FCN-2 (GN4; murine IgG1), and FCN-3 (4H5; murine IgG1) were obtained from Hycult Biotech (Uden, the Netherlands). A second mAb against FCN-3 (334; murine IgG1) was obtained from Enzo Life Sciences (Bruxelles, Belgium). Affinity-purified recombinant human MBL [rMBL at 3.1 mg/ml in 10 mM Tris–HCl with 140 mM NaCl, pH 7.4, and 5 mM EDTA (TBS-E)] (22), recombinant human MASP-1 (rMASP-1 at 0.28 mg/ml), recombinant MASP-2 (rMASP-2) at 3.1 µg/ml (23), and recombinant MASP-3 (0.425 mg/ml), all expressed in HEK cells, and mAbs against MASP-1/3 (4H2A9) and MASP-3 (38:12.3) were a kind gift of Professor J. C. Jensenius (Aarhus University, Aarhus, Denmark). High-performance ELISA buffer (HPE) and poly-HRP-labeled streptavidin (poly-HRP) were obtained from Sanquin (Amsterdam, the Netherlands). 3,5,3′,5′-Tetramethylbenzidine (TMB) was obtained from Merck (Darmstadt, Germany). Mannan and acetylated BSA were obtained from Sigma-Aldrich (St. Louis, MO, USA). Serum from healthy volunteers was obtained with informed consent and prepared as described elsewhere (24). Ethical review and approval were obtained for this study in accordance with the local legislation and institutional requirements (Sanquin Research Medical Ethical Committee). MBL status was determined by genotype and MBL serum levels. All serum aliquots were stored at −80°C until tested.

### Monoclonal Anti-MASP-2 Antibody (mAb 12D12) and MASP-Specific ELISAs

Monoclonal antibodies against pdMBL were obtained by a fusion of spleen cells from a mouse immunized with MBL purified from Cohn fraction III (20). We and others have described that during purification of MBL several other proteins are co-purified (17, 25). During selection of the mAbs, several mAbs appeared to be non-responsive to recombinant MBL (rMBL). These mAbs were further characterized for their reactivity toward MASPs. Briefly, 96-well Nunc Maxisorp microtiter plates were coated overnight with 0.5 µg/ml of different recombinant MASPs in PBS (MASP-1 and MASP-2). All following steps were conducted in HPE. The reactivity of biotinylated mAbs obtained from the original immunization, and control (biotinylated) antibodies (αMASP-1 4H2A9 and αMASP-3 38:12.3) were assessed by titration on the plate. After washing, plates were incubated with 0.01% poly-HRP and developed using 0.1 mg/ml TMB in 0.1 M sodium acetate containing 0.03% (v/v) H_2_O_2_, pH 5.5. Absorbance was measured at 450 nm. In total, 42 non-MBL-binding mAbs were tested for their specificity. mAb 12D12 detected only MASP-2, and not MASP-1 and MASP-3 (Figure S1A in Supplementary Material). As a control for coating the MASPs, we also developed an assay with mAbs against MASP-1/3 (Figure S1B in Supplementary Material).

### MBL Serum Levels and Genotyping

Mannan-binding lectin concentration was measured by ELISA as previously described by Brouwer et al. (16). Briefly, mannan was coated to the solid phase, and biotinylated αMBL-1 was used as detection mAb. After washing, plates were incubated with 0.01% poly-HRP and developed as described earlier. Samples were compared with the mean MBL serum level found in a pool of 3,000 healthy control sera (1.5 µg/ml MBL). MBL status was confirmed using genotyping, as described previously by Frakking et al. (26) In short, a TaqMan assay with specific primers and minor groove binding for each SNP were used, hereby directly amplifying, using both forward and reverse allele-specific primers, the coding polymorphisms.

### Size-Exclusion Chromatography (SEC)

Size-exclusion chromatography was performed in running buffer [veronal-buffered (VB) saline with added 140 mM NaCl]. MBL-deficient serum was reconstituted with rMBL [final concentration 50 µg/ml and incubated at room temperature (RT) for 60 min and, like MBL-sufficient and MBL-deficient sera, double filtered with a 0.2 µM Whatman filter (GE Healthcare, Buckinghamshire, UK)], before 500 µl sample was applied to 23.5 ml, 10 mm × 300 mm Superdex™ 200 10/300 GL (GE Healthcare, Buckinghamshire, UK) prepacked column equilibrated and eluted with running buffer at a constant 0.5 ml/min flow rate. 500 µl fractions were collected in 96-deep well plates and stored at 4°C until tested. The column was calibrated with GE Filtration Calibration Kits (GE Healthcare, Buckinghamshire, UK) according to the manufacturer’s guidelines.

### LP Components Detected by ELISA

All incubations were performed in 100 µl volume at RT, all washes in between incubation steps were done with water. Different fractions, obtained from SEC, were incubated at mannan-coated plates, as described earlier, and incubated for 60 min at RT. After washing, different components (MBL, MASP-1/3, and MASP-2) were detected using specific biotinylated mAbs (αMBL-1, αMASP-1/3 4H2A9, and αMASP-2 12D12) by incubation for 1 h at RT. Finally, poly-HRP [0.01% in VB with added 10 mM CaCl_2_, 2 mM MgCl_2_, 0.3% (v/v) BSA, and 0.02% (v/v) Tween-20 (VB^2+^ B/T)] was added to each well and incubated for 20 min at RT. Plates were developed as described previously.

To circumvent binding of already preformed MBL/MASP complexes and to detect the presence of MASP-1/3 and MASP-2 in MBL-deficient serum, mannan-coated plates were incubated with a fixed amount of rMBL (0.2 µg/ml). Briefly, different fractions were diluted (1:5) in VB saline with added 10 mM CaCl_2_, 2 mM MgCl_2_, 0.3% (v/v), and 0.02% (v/v) Tween-20 (VB^2+^ B/T) and incubated for 1 h at RT. Plates were developed and detected as described earlier. Absorbance was measured at 450 nm and compared with a normal serum pool which was set to 100%.

The functional binding of FCN-1, FCN-2, and FCN-3 was determined by overnight coating with acetylated BSA (5 µg/ml in 0.1 M carbonate buffer, pH 9.6) in 96-well Nunc Maxisorp microtiter plates (Invitrogen, Breda, the Netherlands). After washing, different fractions were diluted 1:50 in VB^2+^ B/T and incubated for 60 min. The binding of FCN was determined by incubation for 60 min at RT with specific biotinylated mAbs: αFCN-1 (AF4209), αFCN-2 (GN4), and αFCN-3 (FCN334) in VB^2+^ B/T. Finally, plates were developed with poly-HRP (0.01% in VB^2+^ B/T) and developed by addition of 0.1 mg/ml TMB I 0.11 M sodium acetate, containing 0.003% (v/v) H_2_O_2_, pH 5.5. Absorbance was measured at 450 nm and compared with a normal serum pool.

## Results

### Elution Profile of MBL-Deficient and MBL-Sufficient Serum

Serum of either MBL-sufficient or MBL-deficient donors was fractionated by SEC to determine the elution profile of MBL and the MASPs. MBL serum levels of MBL-deficient donor were below 0.1 µg/ml and confirmed by genotype (HYPD/LXPA). The MBL-sufficient donor had MBL serum levels of 6.4 µg/ml (genotype HYPA/LYQA). MBL-deficient serum was completely restored in LP functionality following *in vitro* substitution with rMBL, indicating the presence of normal levels of LP components (data not shown).

**Figure 1:**
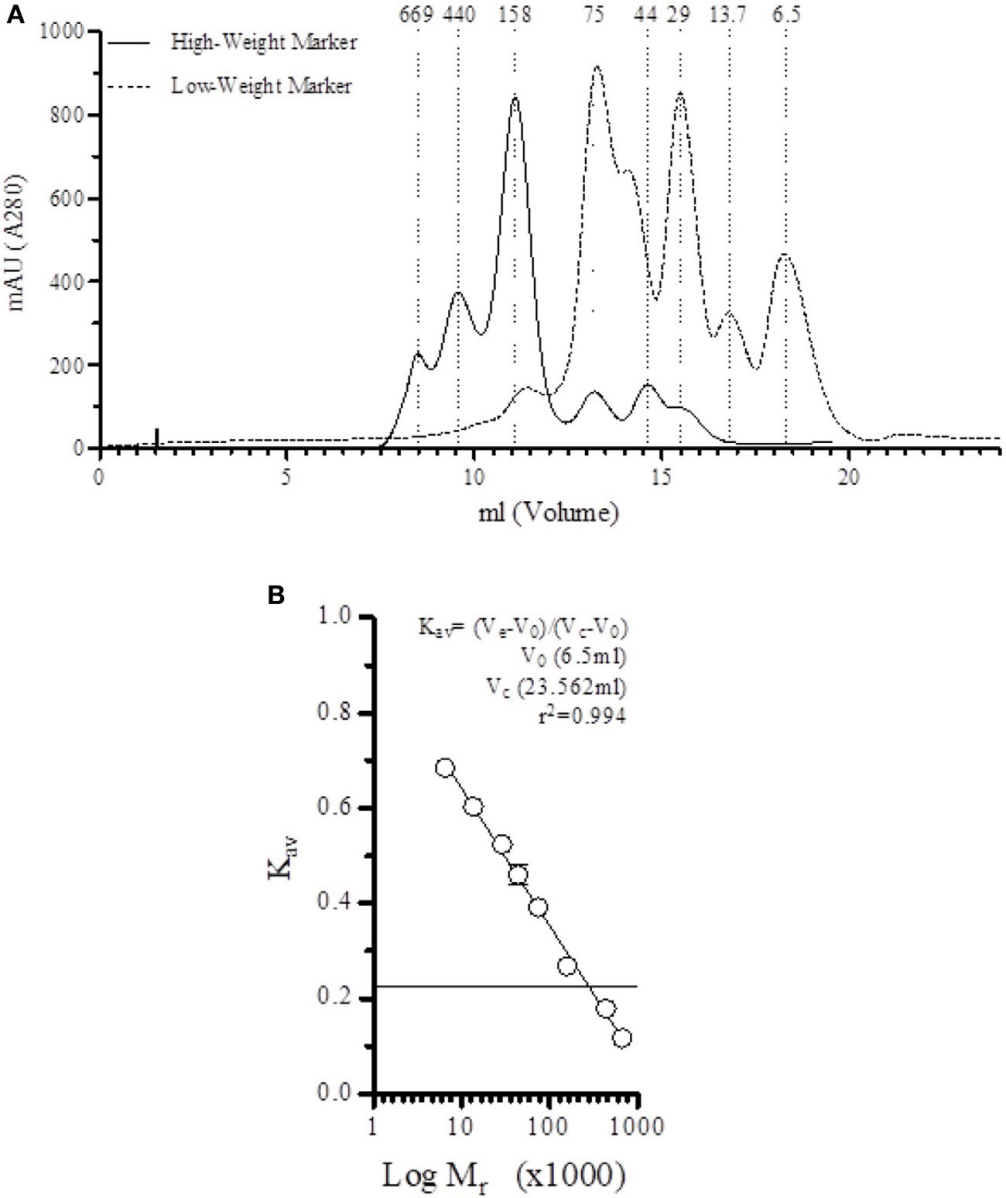
Direct correlation between elution fraction and size. **(A)** Elution profile of low-weight and height-weight markers on the Superdex™ 200 10/300 GL in veronal-buffered saline (1 M NaCl, pH 7.4). **(B)** A strong correlation (*r*^2^ = 0.994) is found between the elution volume and the marker size.

Before fractionation of serum, the column used in the SEC was equilibrated using running buffer and calibrated with low- and high-weight markers (Figure 1A). A direct correlation (*r*^2^ = 0.994) between elution volume and marker size was calculated (Figure 1B), allowing us to correlate the relative size of the protein based on the elution profile.

**Figure 2:**
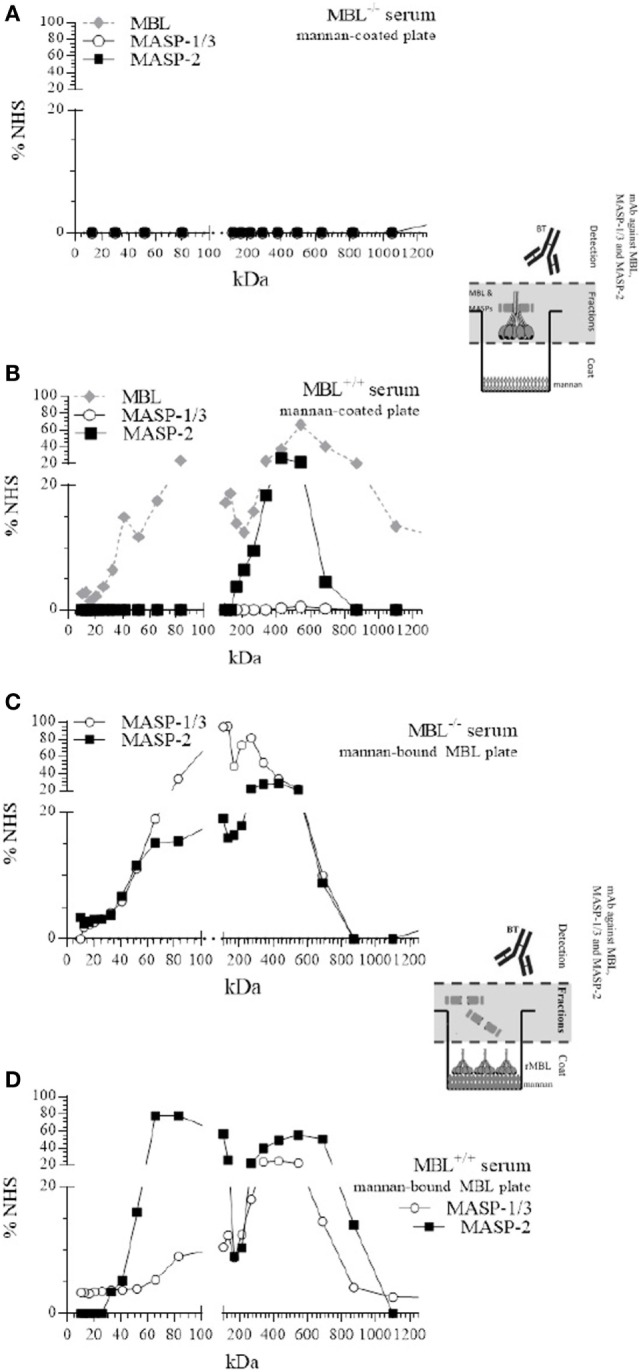
Size-exclusion chromatography (SEC) of mannan-binding lectin (MBL)-deficient and MBL-sufficient serum. SEC of MBL-deficient serum **(A,C)** or MBL-sufficient serum **(B,D)**. Serum was fractionated on a Superdex™ 200 10/300 GL column **(A,C)**. Fractions were tested for the presence of MBL, or MBL–MBL-associated serine protease (MASP)-1/3, MBL–MASP-2 complexes on mannan-coated plates and compared with a pool of normal human sera (NHS). **(B,D)** Mannan-coated plates were pre-incubated with fixed amount of recombinant MBL, and followed by the different fractions of MBL-deficient or MBL-sufficient serum, and the formation of new MBL–MASP-1/3 or MBL–MASP-2 complexes was detected and compared with a pool of NHS.

First, all fractions were incubated on mannan-coated plates, and bound MBL and complexed MASPs were detected. As expected, after fractionation of MBL-deficient serum, no LP components were detected on mannan-coated plates (Figure 2A). Fractionation of MBL-sufficient serum showed the presence of MBL and MBL/MASP-2 complexes binding to the mannan-coated plate (Figure 2B). Mannan-bound MBL was mainly observed at 550 kDa and around 80 kDa, corresponding to a polymeric and a trimer of the 32-kDa MBL protein. A single peak of complexed MBL/MASP-2, around 430 kDa, was observed, indicating that MASP-2 only associated with higher-order oligomers. We did not find any MASP-1/3 in the higher polymeric forms of MBL being not detected on mannan-coated plates in these experimental conditions.

### Redistribution of MASPs

To investigate whether MASP-1/3 and MASP-2 were able to redistribute from serum and associate to mannan-bound rMBL, all fractions were incubated on the mannan coat, which was first saturated with rMBL. Detection with mAb against MASP-1/3 and MASP-2 revealed that both MASP-1/3 and MASP-2 from the MBL-deficient serum were able to associate with mannan-bound rMBL (Figure 2C). MASP-1/3 that associated with rMBL on the plate was derived from fractions containing proteins with different molecular sizes, i.e., at around 100–130 and 210–260 kDa. MASP-2 showed a similar elution profile with a peak around 440 kDa. Both MASPs were apparently also present in their monomeric free form, not bound to any collectin including MBL, as indicated by the presence of the protein in fractions corresponding to a molecular size of 70 kDa.

MBL-associated serine protease-2 from the MBL-sufficient serum that associated with rMBL on the plate was derived from fractions containing high-molecular weight proteins (peak around 540 kDa) in which the polymeric MBL was present, and from fractions containing lower molecular weight proteins (65–84 kDa), corresponding to non-complexed monomers of MASP-2. In this serum, MASP-1/3 showed a more diffuse elution pattern with a peak around 430 kDa, which was at a higher molecular weight compared with that in MBL-deficient serum and could correspond to serum fractions containing FCNs. Like MASP-2, MASP-1/3 was also found in a fraction of MBL-sufficient serum with a relative size corresponding to a non-complexed, free monomeric form (Figure 2D).

### Restoration of MBL-Deficient Serum by rMBL

The addition of rMBL to MBL-deficient serum can completely restore the LP functionality *in vitro* as shown by a recovery of C4-deposition (18). To investigate whether rMBL associates with free MASPs in fluid phase or MASPs that are in complex with other collectins (for instance, FCNs) may redistribute to rMBL, MBL-deficient serum was reconstituted with rMBL and subsequently fractionated. rMBL contains primarily trimeric (225 kDa), tetrameric (300 kDa), and pentameric (375 kDa) oligomers (16, 27). All fractions were incubated on a mannan-coated plate and tested for the presence of MBL–MASP complexes.

**Figure 3:**
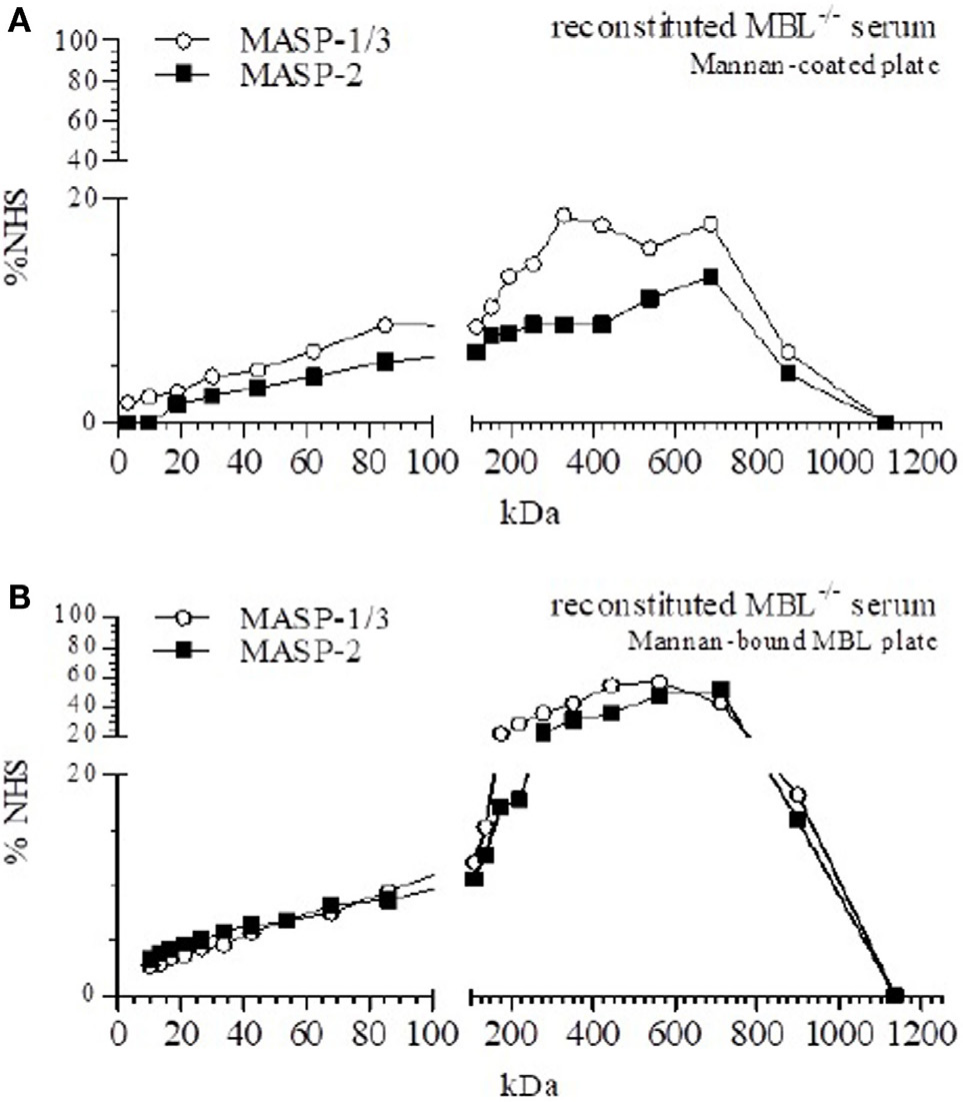
Size-exclusion chromatography (SEC) of mannan-binding lectin (MBL)-reconstituted MBL-deficient serum. SEC of MBL-deficient serum reconstituted to 50 µg/ml MBL. **(A)** Fractions were tested for the presence of MBL–MBL-associated serine protease (MASP)-1/3 and MBL–MASP-2 complexes on mannan-coated plates. **(B)** Mannan-coated plates were pre-incubated with MBL, and followed by the different fractions of reconstituted MBL-deficient serum, and the formation of MBL–MASP-1/3 and MBL–MASP-2 complexes was detected.

Both MBL/MASP-1/3 and MBL/MASP-2 were detected in fractions containing high-molecular weight proteins (Figure 3A), indicating that in fluid-phase rMBL associated with MASP-1/3 and MASP-2. MBL/MASP-1/3 complexes were found in a diffuse pattern, but the most prominent peaks were around 350 and 710 kDa. MBL/MASP-2 complexes showed a similar pattern with a main peak around 710 kDa.

To determine the origin of the MASPs that associated with rMBL in fluid phase, all fractions were also analyzed on mannan-coated plates saturated with rMBL (Figure 3B). Both MASP-1/3 and MASP-2 were found in a distinct peak around a relative size of 440–560 kDa for MASP-1/3 and around 560–710 kDa for MASP-2. In contrast to the previously observed double peak for MASP-1/3, we now observed a single peak at a higher molecular weight with a noted absence of the non-complexed form of MASP, suggesting that free soluble MASP-1/3 and MASP-2 associated with rMBL once added. However, our experimental setup was not able to distinguish between heterocomplexes of MASP-1/3 and MASP-2 within a single MBL protein or separate complexes of either MBL-associated MASP-1/3 or MBL-associated MASP-2.

### FCN/MASP Complexes

We hypothesized that formation of MBL/MASP complexes more readily happens when MBL is bound to mannan, hereby attracting MASPs from FCN/MASP complexes (Figure 2). We therefore investigated the presence of different FCNs in the fractions of MBL-deficient and reconstituted MBL-deficient serum. FCN-1 was not detected in any of the fractions.

**Figure 4:**
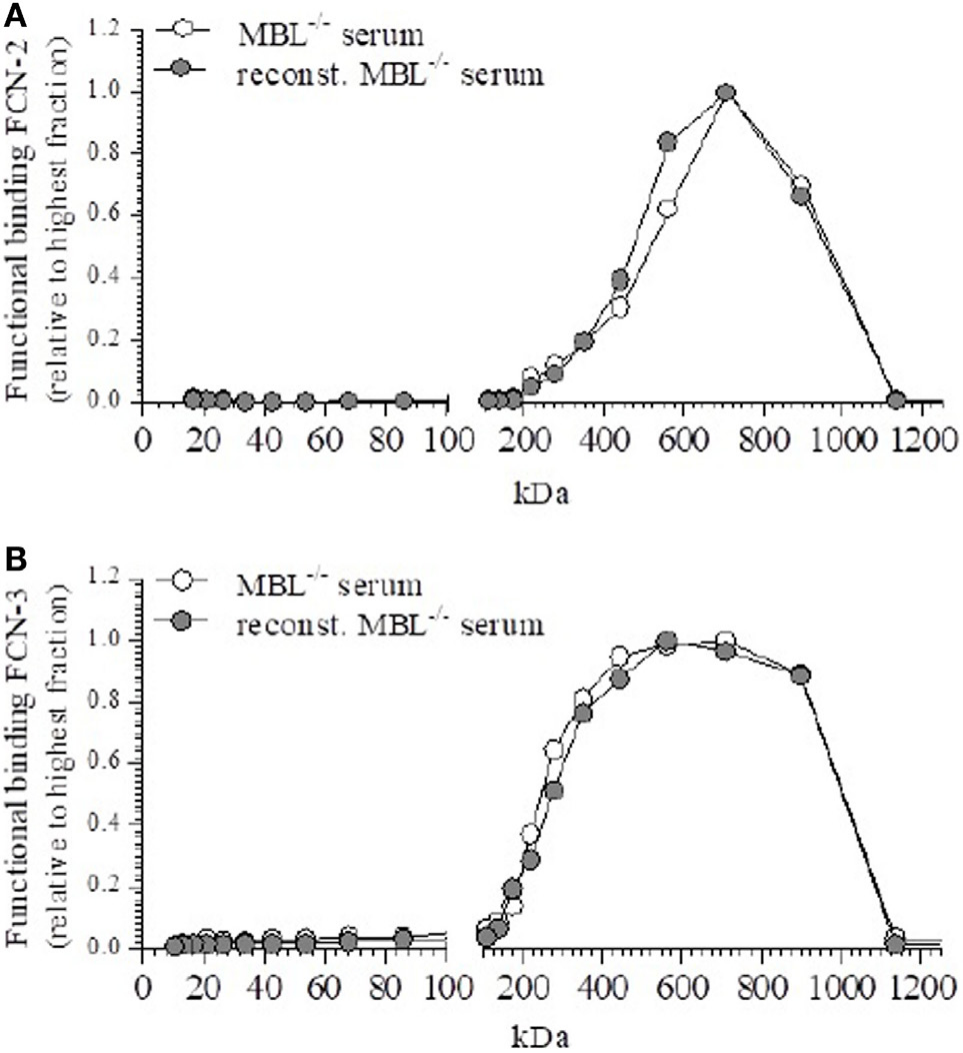
Functional binding of ficolin (FCN)-2 and FCN-3. Effect on the distribution of FCN-2 and FCN-3 after reconstitution to 50 µg/ml mannan-binding lectin (MBL). **(A)** Fractions were tested for the ligand binding of FCN-2 before and after reconstitution. **(B)** Fractions were tested for the ligand binding of FCN-3 before and after reconstitution.

The fractions of MBL-deficient serum and reconstituted MBL-deficient serum showed the functional binding of FCN-2 to acetylated BSA as a peak around 540 kDa (Figure 4A), which did not alter after rMBL-reconstitution. Similar observations were made for FCN-3 (Figure 4B). Neither FCN-2 nor FCN-3 showed a different elution profile after reconstitution with rMBL, suggesting that the detected MASPs, in the mannan-bound MBL assay, are not derived from FCN/MASP complexes but were unbound MASPs (low molecular fraction) that reacted with the ligand-bound MBL.

## Discussion

In this study, we investigated the association of rMBL with MASPs in MBL-deficient serum. Our data demonstrated the unexpected presence of free non-complexed MASP-1/3 and MASP-2 that could associate to fluid-phase rMBL when added to MBL-deficient serum.

MBL-associated serine protease-1/3 and MASP-2 can associate with different LP PRMs. It has been hypothesized that MASPs are in equilibrium and can freely associate with the different available PRMs which act as carrier proteins (23, 28). We hypothesized that upon binding of these PRMs to their ligands the equilibrium of MASP binding would shift in favor of the ligand-bound protein, resulting in the formation of PRM/MASP complexes to initiate the complement activation cascade. *In vitro* substitution of MBL-deficient serum with rMBL restored the C4-deposition on mannan-coated plates, indicating the formation of functional rMBL/MASP complexes. Our findings showed the ability of mannan-bound rMBL to form new rMBL/MASP complexes in MBL-deficient serum and MBL-sufficient serum. Both MASP-1/3 and MASP-2 were found to associate with mannan-bound rMBL. The relative size of the newly formed complexes suggested that MASPs are present as free non-complexed proteins as well as in a bound form, being associated with different LP complexes. A likely source of these LP complexes would be FCN-2 and FCN-3 in MBL-deficient serum. This is different form previous published reports showing the tendency of MASPs to form a dimeric molecule following analysis of recombinant proteins or complexes (29–31), which could be related to the origin of the proteins analyzed.

The elution profile of MBL-sufficient serum showed immediate similarity with the previously published results of Dahl et al. (4) These authors described different polymeric forms of MBL, ranging from relative sizes of 275 kDa and smaller (MBL-I), to 345–580 kDa (MBL-II), 580–900 kDa (MBL-III), and 900 kDa and above (MBL-IV). Although we were able to detect MASP-1 in unfractionated MBL-sufficient serum, the different fractions obtained from SEC after fractionation showed no detectable signal on mannan-coated plates. We confirmed that MASP-2 primarily associated with MBL-II in MBL-sufficient serum. After reconstitution of MBL-deficient serum, we were able to detect both MASP-1/3 and MASP-2 on mannan and mannan-bound MBL plates primarily in fractions corresponding to higher sizes, between 200 and 900 kDa. This suggests that an association takes place between other LP complexes and rMBL in fluid phase. We were unable to detect the presence of FCN-1 in our fractions, which was to be expected, given the low serum concentration of FCN-1 and further dilution during fractionation (32, 33). In contrast to FCN-1, higher-order oligomers of FCN-2 were detected in fractions corresponding to a relative size of 710 kDa, and FCN-3 oligomers were detected in a broad range of sizes 400–900 kDa. Although Ohashi and Erickson (34) and Munthe-Fog et al. (35) have reported even larger sizes around 800–870 kDa, our column has a lower discriminatory sensitivity for the very high-molecular weights. The distribution patterns of FCN-2 and FCN-3 are similarly between MBL-deficient serum and reconstituted MBL-deficient serum, supporting the hypothesis of MASP-2 preferentially binding to a ligand-bound protein. This is also supported by Megyeri et al. (36) who showed an increased yield of MBL/MASP complexes following addition of recombinant MBL and subsequent purification using mannan-sepharose. The pattern of FCN-2 and FCN-3 appear to correspond to the observed pattern of newly formed MASP complexes on mannan-bound MBL, we were unable to observe a relative change in size.

The presence of a non-complexed form of MASP-2 and MASP-1/3 provides a new mechanism of restoration of the LP activity following MBL reconstitution. This non-complexed free form of MASP-2 and MASP-1/3 could no longer be detected following addition of rMBL to MBL-deficient serum, leading to the formation of a new complex with a larger molecular size indicating a redistribution of MASPs, which was suggested before by Megyeri et al. (36). Whether these different MASPs consist of homodimers or heterodimers before association with oligomeric MBL is still debated (6, 28, 37, 38).

Early MBL substitution studies in MBL-deficient patients have shown the safety of infusion of pdMBL (11), but a limited restoration of the LP functionality was observed despite reaching high plasma MBL levels (16). Analysis of pdMBL showed high levels of associated pre-activated MASP-2 (27), which is rapidly inactivated upon *in vivo* infusion by natural inhibitors such as C1-inhibitor (17). The MBL-associated MASPs in the pdMBL product are rapidly cleared within 24 h after substitution with limited restoration of the LP (16). By contrast, rMBL has been shown to efficiently restore the LP functionality as indicated by C4-deposition both *in vitro* (18) and *in vivo* (36). The potential therapeutic value of MBL replacement depends on the potential to form activating complexes with MASPs that are functional upon ligand binding. Our study showed the presence of a natural non-bound form of MASP-2 in serum to explain the mechanism by which rMBL can restore LP functionality in MBL-deficient patients. These rMBL–MASP complexes are not yet activated, in contrast to the pre-activated MASPs bound to MBL due to affinity chromatography purification (17, 25). Our results indicate that rMBL—upon its formation of functional complexes after infusion—will not be promptly inhibited and cleared as we had previously observed for pdMBL (17). Both the fact that rMBL substitution was well-tolerated in a phase I trial (39) and the presence of non-bound forms of MASPs able to associate with rMBL and restore LP functionality provide a rationale to consider new clinical rMBL substitution studies in carefully selected patients.

## Author Contributions

Study concept and design; drafting of manuscript: MK, DW, and TK. Acquisition of data: MK, AK, and GM. Statistical analysis of data: MK, AK, and DW. Interpretation of data: MK, AK, DW, and TK. Critical revision of the manuscript for important intellectual content: all the authors.

## Conflict of Interest Statement

The authors declare that the research was conducted in the absence of any commercial or financial relationships that could be construed as a potential conflict of interest.

## Supplementary Material

Figure S1 Specific monoclonal antibody (mAb) against MBL-associated serine protease (MASP)-2. Plates were directly coated with recombinant proteins, respectively, rMASP-1 (○), recombinant MASP-2 (rMASP-2) (■), and rMASP-3 (□). Coated proteins were detected using a titration of specific biotinylated mAbs against MASP-1/3 (4H2A9) (36), against MASP-2 (12D12) or against MASP-3 (38:12-3) (36). **(A)** mAb 12D12 binds only to MASP-2 and does not show cross-reactivity toward the other serine protease. **(B)** mAb 4H2A9 is able to detect both MASP-1 and MASP-3 bound to the plate, by binding to a shared epitope (40).

Figure S2 Reconstitution with recombinant MBL (rMBL) restores lectin pathway functionality. Plates were directly coated with mannan and a serum dilution of reconstituted mannan-binding lectin (MBL)-deficient serum with 50 μg/ml rMBL (●), MBL-deficient serum (○), or MBL-sufficient serum was tested for their C4-converting function.
