# Supplementary material for: Substitution of Mannan-Binding Lectin (MBL)-Deficient Serum With Recombinant MBL Results in the Formation of New MBL/MBL-Associated Serine Protease Complexes
Source: Front Immunol. 2018 Jun 27;9:1406. doi: 10.3389/fimmu.2018.01406 (PMC6030254; doi:10.3389/fimmu.2018.01406)
Supplement: Figure S1 — Specific monoclonal antibody (mAb) against MBL-associated serine protease (MASP)-2. Plates were directly coated with recombinant proteins, respectively, rMASP-1 (○), recombinant MASP-2 (rMASP-2) (■), and rMASP-3 (□). Coated proteins were detected using a titration of specific biotinylated mAbs against MASP-1/3 (4H2A9) (36), against MASP-2 (12D12) or against MASP-3 (38:12-3) (36). (A) mAb 12D12 binds only to MASP-2 and does not show cross-reactivity toward the other serine protease. (B) mAb 4H2A9 is able to detect both MASP-1 and MASP-3 bound to the plate, by binding to a shared epitope (40). [file Image_1.PDF]

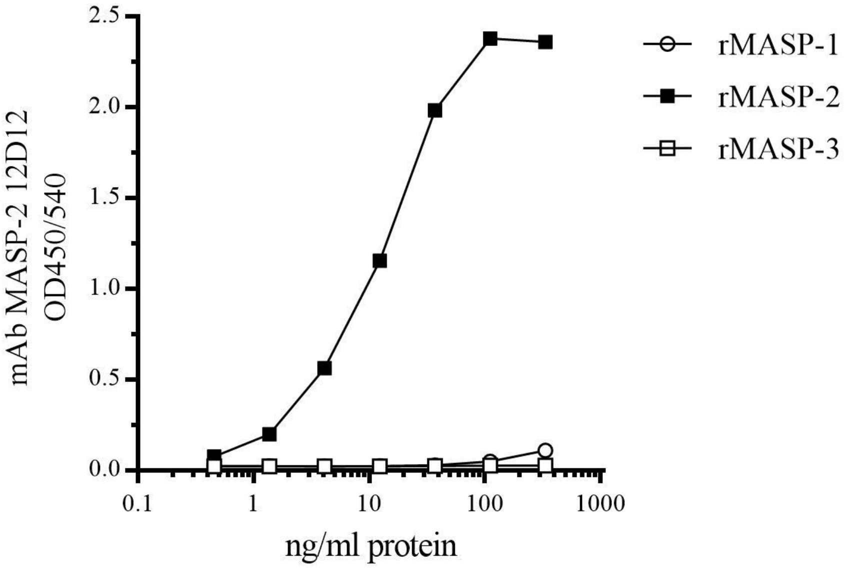

A.

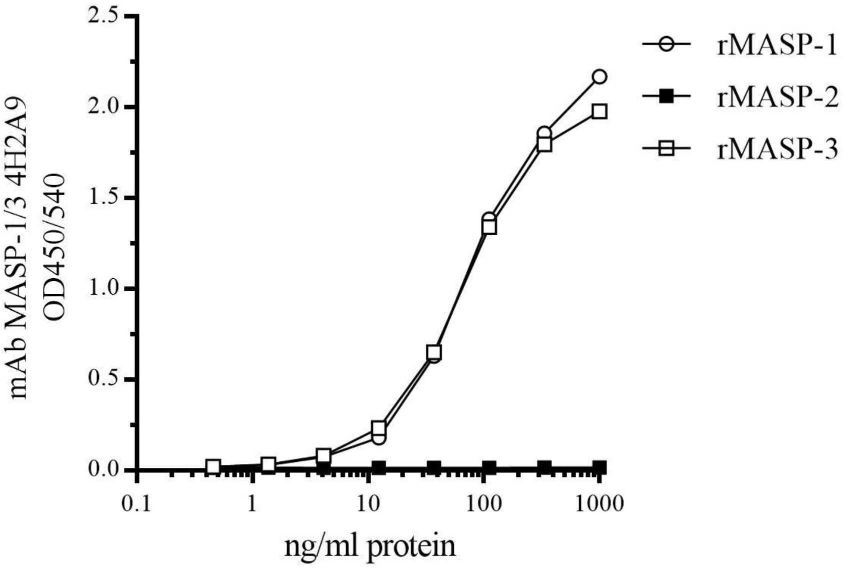

B.

Supplemental Figure 1. Specific monoclonal antibody against MASP-2.

Plates were directly coated with recombinant proteins, respectively rMASP-1 (○), rMASP-2 (■) and rMASP-3 (□). Coated proteins were detected using a titration of specific biotinylated monoclonal antibodies (mAb) against MASP-1/3 (4H2A9)(38), against MASP-2 (12D12) or against MASP-3 (38:12-3).(38) ***(A.)*** mAb 12D12 binds only to MASP-2, and does not show cross-reactivity towards the other serine protease ***(B.)*** mAb 4H2A9 is able to detect both MASP-1, and MASP-3 bound to the plate, by binding to a shared epitope.(38)
