# Supplementary figures and images for: Substitution of Mannan-Binding Lectin (MBL)-Deficient Serum With Recombinant MBL Results in the Formation of New MBL/MBL-Associated Serine Protease Complexes
Source: Front Immunol. 2018 Jun 27;9:1406. doi: 10.3389/fimmu.2018.01406 (PMC6030254; doi:10.3389/fimmu.2018.01406)

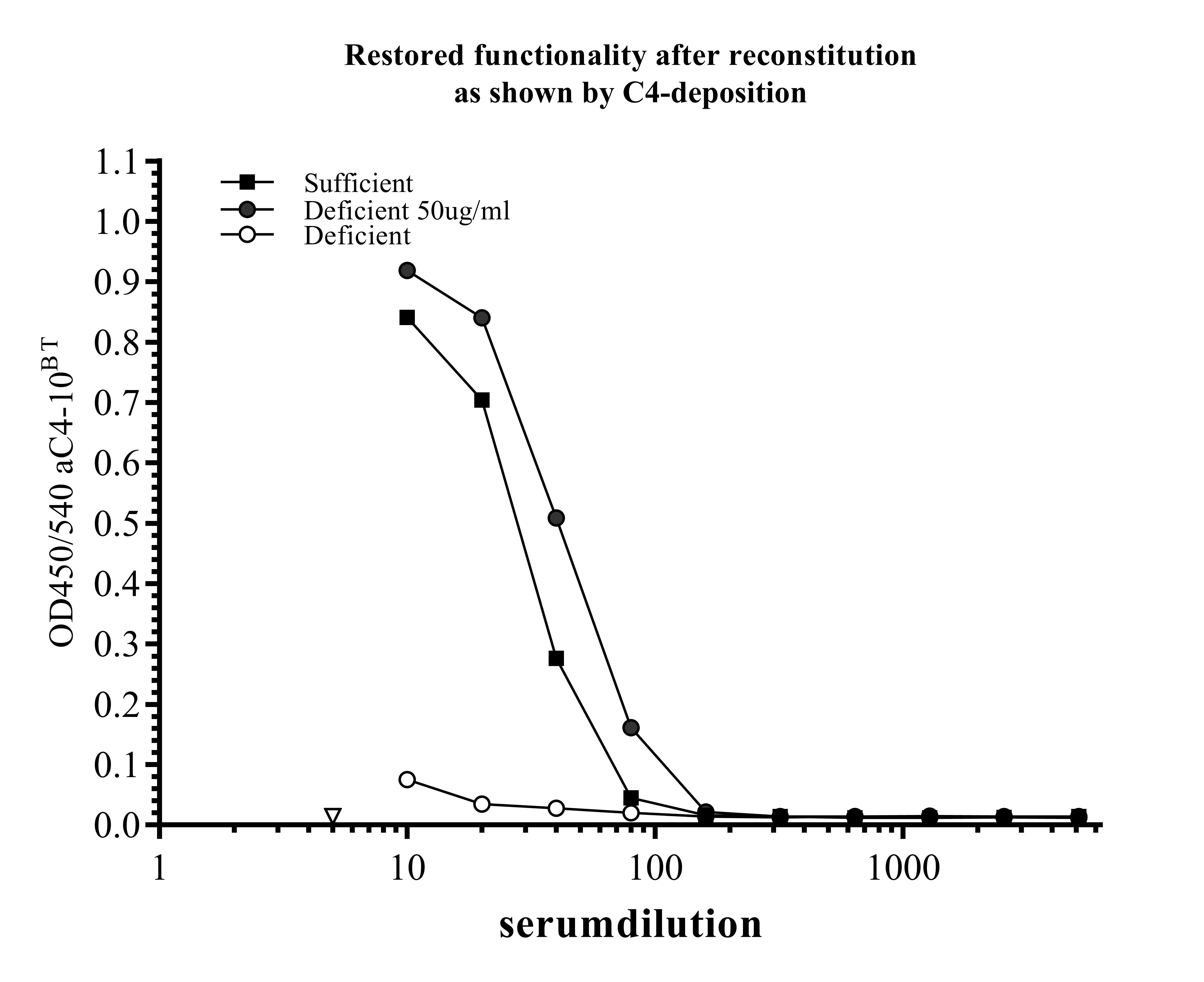

Supplement: Figure S2 — Reconstitution with recombinant MBL (rMBL) restores lectin pathway functionality. Plates were directly coated with mannan and a serum dilution of reconstituted mannan-binding lectin (MBL)-deficient serum with 50 μg/ml rMBL (●), MBL-deficient serum (○), or MBL-sufficient serum was tested for their C4-converting function. [file Image_2.jpg]
